# The use of Foley catheter tamponade for bleeding control in penetrating injuries

**DOI:** 10.1186/s13049-021-00975-2

**Published:** 2021-12-04

**Authors:** Nicolas Beysard, Mathieu Pasquier, Tobias Zingg, Pierre-Nicolas Carron, Vincent Darioli

**Affiliations:** 1 Department of Emergency Medicine, Lausanne University Hospital, 1011 Lausanne, Switzerland; 2 Department of Emergency Medicine, Lausanne University Hospital and University of Lausanne, 1011 Lausanne, Switzerland; 3 Department of Surgery, Lausanne University Hospital and University of Lausanne, 1011 Lausanne, Switzerland

**Dear Editor,**

With great interest, we have read the review article of Simpson et al. [1] on the current evidence and recommendations for the pre-hospital management of penetrating neck injuries (PNI). We would like to comment on the use of Foley catheters to achieve bleeding control from non-compressible penetrating injuries.

In their scoping review, the authors appropriately focus on the application of haemostatic dressings to achieve external bleeding control, but they also suggest to consider Foley catheters as an additional measure to manage bleeding from PNI. Their review process identified a single case series describing the use of Foley catheters in 11 military patients [2]. Some important additional references should be cited to support the use of this adjunct. A recent article by Himmler et al. described the use of this technique in 29 civilian patients actively bleeding from penetrating torso trauma in a single major urban centre in South America [3]. The 24-h mortality was 3.4% and the 30-day mortality was 17.9%. Moreover, in 2020 Scriba et al. described a cohort of 95 patients with PNI in a South-African level I trauma centre, treated with Foley catheters [4]. Bleeding control with haemodynamic stabilisation was achieved in 92 patients (96.8%).

We recently used a Foley catheter to manage a stab wound to the left anterior neck (zone 1) in a 26 year-old male. Police arrived shortly after the incident but couldn’t stop the bleeding with external compression with gauzes. After the arrival of the pre-hospital emergency physician, a 16 French Foley catheter was inserted into the wound and the balloon was inflated with 20 ml of saline solution (Figs. 1 and 2), allowing the bleeding to stop immediately. Definitive surgical management revealed a 4–5 cm zone 1 neck wound, with injuries to the anterior jugular veins.

**Fig. 1:**
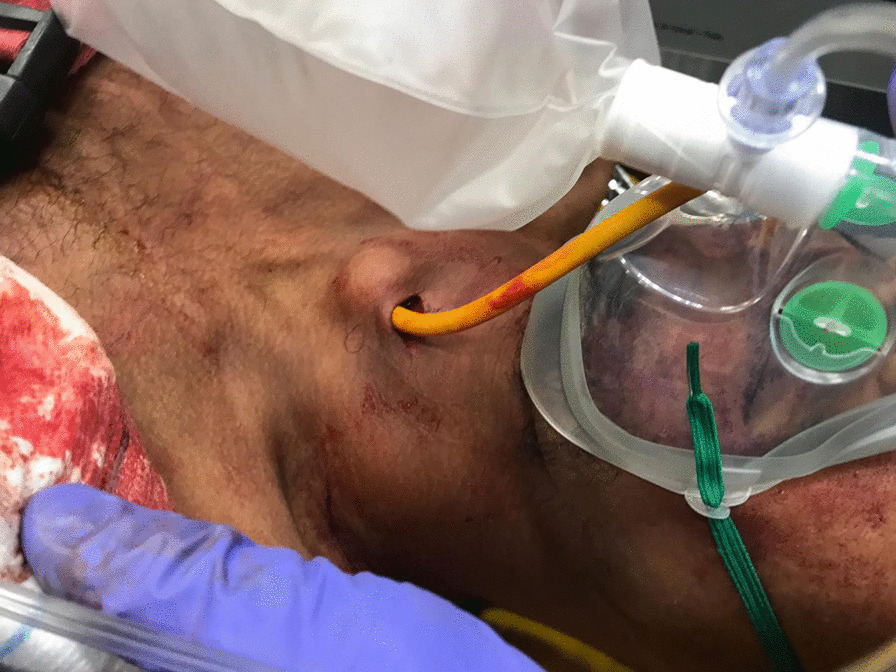
Foley catheter inserted into zone 1 wound

**Fig. 2:**
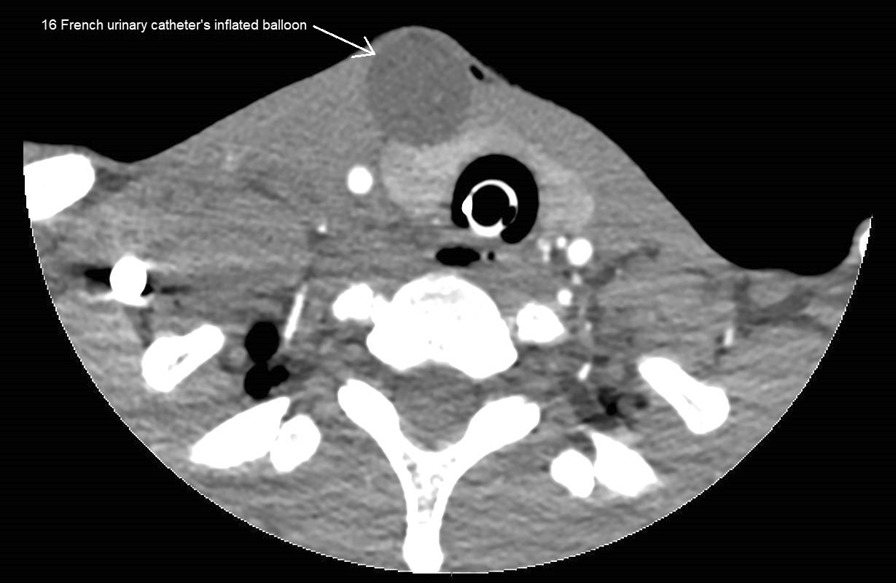
Cervical computed tomography scan with Foley catheter inflated

External bleeding control is a well-known challenge for both pre-hospital and hospital teams, as illustrated by the military c-ABC approach of trauma patients [5]. The main conclusion of the scoping review conducted by Simpson et al. is that the currently available evidence pertaining to the prehospital management of PNI is scarce, and essentially based on observational data with low sample sizes. While we agree with the authors that a consensus on the pre-hospital management of bleeding from non-compressible penetrating injuries is needed, we suggest that Foley catheter balloon tamponade should be emphasized as a valuable option.

## Data Availability

Not applicable.

## Abbreviation

PNI: Penetrating neck injuries
