# Supplementary material for: The use of Foley catheter tamponade for bleeding control in penetrating injuries
Source: Scand J Trauma Resusc Emerg Med. 2021 Dec 4;29:165. doi: 10.1186/s13049-021-00975-2 (PMC8642887; doi:10.1186/s13049-021-00975-2)
Supplement: Related texts

# Reply: Response to letter entitled ‘The use of Foley catheter tamponade for bleeding control in penetrating injuries’

**Authors:** Christopher Simpson, Harriet Tucker, Anthony Hudson

**Affiliations:** 4 grid.464688.0 0000 0001 2300 7844 Emergency Department, St George’s Hospital Trust, Blackshaw Rd, Tooting, London, SW17 0QT UK; 5 Air Ambulance Kent Surrey Sussex, Redhill Airfield, Redhill, Surrey, RH1 5YP UK

**Dear Editor,**

I would like to thank Dr. Beysard and colleagues for bringing these other two published examples of Foley catheter balloon tamponade use to our attention, along with their recent use of this method. We would welcome their experience and input as part of the proposed Delphi project, along with that of all clinicians with experience managing these injuries. Through combined experience and literature assimilation we hope to generate the most clinically valuable consensus statement possible.
